# A Comparative Study between Scanning Devices for 3D Printing of Personalized Ostomy Patches

**DOI:** 10.3390/s22020560

**Published:** 2022-01-12

**Authors:** Sofia Zahia, Begonya Garcia-Zapirain, Jon Anakabe, Joan Ander, Oscar Jossa Bastidas, Alberto Loizate Totoricagüena

**Affiliations:** 1 eVIDA Research Group, University of Deusto, 48007 Bilbao, Spain; mbgarciazapi@deusto.es (B.G.-Z.); oscar.jossa@deusto.es (O.J.B.); 2 LEARTIKER S.COOP, 48270 Markina, Spain; janakabe@leartiker.com (J.A.); 1995ander@gmail.com (J.A.); 3 Servicio de Cirugía General y del Aparato Digestivo, OSI Bilbao-Basurto, Osakidetza, 48013 Bilbao, Spain; alberto.loizatetotoricaguena@osakidetza.eus

**Keywords:** additive manufacturing, ostomy, medical devices, 3D scanners

## Abstract

This papers presents a comparative study of three different 3D scanning modalities to acquire 3D meshes of stoma barrier rings from ostomized patients. Computerized Tomography and Structured light scanning methods were the digitization technologies studied in this research. Among the Structured Light systems, the Go!Scan 20 and the Structure Sensor were chosen as the handheld 3D scanners. Nineteen ostomized patients took part in this study, starting from the 3D scans acquisition until the printed ostomy patches validation. 3D mesh processing, mesh generation and 3D mesh comparison was carried out using commercial softwares. The results of the presented study show that the Structure Sensor, which is the low cost structured light 3D sensor, has a great potential for such applications. This study also discusses the benefits and reliability of low-cost structured light systems.

## 1. Introduction

Colorectal cancer is one of the most frequent tumors and the second in terms of mortality worldwide, and the second most detected type of cancer in the Basque Country, with an 18% of the total amount [1]. The treatment of colon and rectal cancer usually requires the aperture of a stoma, which consists in an opening in the skin of the abdomen (stoma) to externalize a part of the digestive or urinary tract, which allows its contents to be evacuated [2,3]. In a third-level hospital such as the Basurto University Hospital, more than 50 stomatal opening surgeries are performed annually.

The rate of complications related to ostomies (colostomies, ileostomies or urostomies) is highly variable, between 30 and 90% [4,5,6]. The most frequent complication is the change in the configuration of the abdominal perimeter of the patient after the surgery, generated by the variations in their weight and the practice of sports. Additionally, due to the natural intra-abdominal pressure, the orifice tends to increase in size giving rise to parastomal hernias [7,8]. All these alterations mean that on numerous occasions, the commercial ostomy patches, that cover the stoma, do not adapt adequately to the skin of the abdomen, and they detach easily, causing significant problems for the patient.

**Figure 1:**
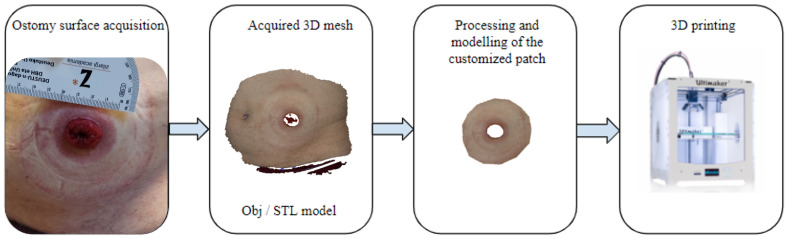
Pipeline illustrating the main steps for the creation of the customized ostomy patches.

Being able to design personalized ostomy patches adapted to the shape of the abdominal wall of each patient, could prevent complications and improve the quality of life of these patients. In this context, INTELOST, a project funded by the Basque Government (ELKARTEK 2019 [9]) aims at developing intelligent patches for ostomy bags, customized to the anatomy of each patient through 3D printing and smart technology, as shown in Figure 1. By incorporating sensors that allow measuring variables of interest from patients, which in the future could be transmitted wirelessly to a mobile device, the designed system will be able to process the acquired data in real time through a cloud computing system with artificial intelligence. The present study presents a comparative study of the 3D scanners used to acquire the 3D meshes of the regions of interest from each patient using images obtained by “low-cost” scanning systems versus conventional “DICOM” scanning techniques.

Medical 3D printing is an innovative technology which is gaining more applications and utilities inside the medical field. The use of 3D printing inside the medical field can have different objectives: preoperative planning and surgical treatment analysis, development of non-bioactive prosthesis or implants, development of bioactive and biodegradable scaffolds for tissue engineering and research of directly printing functional tissues or organs [10,11,12,13]. At the beginning, the typical biomaterials to be used for medical 3D printing were nonfunctional polymers, but with the emergence of biocompatible polymers and fields like tissue engineering, 3D printing applications are getting more interested, as recently reported in a variety of comprehensive literature reviews [14,15,16]. 3D printing polymeric materials for medical applications offers a series of advantages like the diversity of polymer material characteristics, properties and processing methods. 3D printing is a desirable manufacturing process as it offers the possibility to generate complex geometries and architectures that are not possible with conventional manufacturing processes. Moreover, polymer 3D printing allows the printing of either flexible materials or rigid, or biodegradable materials for tissue regeneration applications [17].

The medical field requires geometrically accurate and high-resolution medical images for a precise measurement and diagnosis [18]. Different studies have incorporated the use of 3D scanners in the medical field, being used mainly in face and head scanning [18,19,20,21,22,23]. Crowe S et al. [18] used the Artec Leo structure light scanner for two purposes. The designing and production of radiotherapy medical devices and the synthesis of pseudo-CT datasets for advanced applications. Four objects of different colours and dimensions were scanned. Subsequently, neck, face and head scanning of 26 healthy volunteers were performed. Geometry accuracy was assessed by obtaining the Hausdorff distance between 3d scanning devices and CT images acquired using Siemens Somaton. The results showed minimal deviations between phantom models using 3D scanning by Artec Leo scanner and CT scanner. Similarly, Zhao YJ et al. [22] applied 3D scanning in the assessment of tissue facial disfigurements of 10 patients. They achieved an accuracy of 0.43 ± 0.05 mm using FaceSCAN3D, taking as reference a laser-based system. Artec Eva was used by Modabber A et al. [23] to scan the faces of 41 patients. They stated that it took them longer to scan using the Artec Eva than the FaceSCAN3D scanner. Shah PB et al. [21] analyzed the difference between three types of 3D scanners for scanning the face and head of 10 voluntary participants. The scanners used were Cyberware 3030 colour scanner, Artec Eva 3D scanner and Structure Sensor ST01 mode. Ter Braak TP et al. [24] used the Structure Sensor for the forearm scanning of 24 volunteers. For the assessment of the scanning process, they measured the accuracy by calculating the Pearson’s correlation between the average direct and digital measures obtained for the 3D scanner. They have concluded that the Structure Sensor showed to be a reliable scanner, showing excellent inter-rater reliability with a difference in ICC of 0.001, and is reproducible for the forearm dimensions scanning.

No comparisons were made in terms of quantitative measures with the published works in the literature as no studies that involve ostomy-related 3D scanning were found. However, we discuss some limitations and common advantages among our and other studies (mostly face and head scanning) in the use of 3D scanning in the medical field.

Crowe S et al. [18] mention the advantage of wireless connection and no required previous training using Artec Leo scanner as well as the Structure Sensor in our study. Furthermore, they highlighted the feedback advantages of the screen for the operator, like our study with the Go!scan 20 scanner. Shah PB et al. [21] mentioned that Artec Eva has a friendly and useful software for data processing. Artec Eva, similarly to the Structure Sensor, does not need prior homing calibration. Knoops PG et al. [19] remarked that although the Structure Sensor is not very accurate for some applications, it has the advantage of simple usability and portability, as mentioned in the present study.

Regarding the limitations, different aspects were considered, such as the duration of the scanning sessions, device weight, high level of expertise using the scanners and difficulties to scan some areas of the body. Crowe S et al. [18] took up to 50 min for the session scanning, more than our study that included two scanners and the removal of the first stoma patch and dressing the new one after completing the scanning. The weight of the Artec Leo scanner (2.6 kg) represented discomfort for the operators in the longer sessions, similarly to Go!scan 20, unlike the Structure Sensor. Furthermore, Crowe S et al. [18] and Shah PB et al. [21] mentioned difficulties to scan data in shadowed regions (e.g., areas behind the ear), which also happened in the present study in zones hidden by stomach fat using Go!scan20. The Structure Sensor did not have any problems with these zones. Secher JJ et al. [25] found difficulties scanning different facial expressions or changes in the face position. We had the same problem in stomach areas using Go!Scan 20, when the patients moved during the scanning. Additionally, Shah PB et al. [21] found limitations using the Artec Eva 3D scanner, remarking that this sensor needs a high level of expertise before using it.

The remaining sections of this paper are provided as follows. Section 2 presents the materials and methods used to conduct this research. Section 3 shows the results along with discussion. Finally, Section 4 highlights the conclusion of this study.

## 2. Materials and Methods

### 2.1. Participants

The population included in this study is part of the INTELOST project aims at developing intelligent patches for ostomy meshes, customized to the anatomy of each patient through 3D printing and intelligent technology. The protocol of this pilot study was assessed and approved by the Basque Country Ethics Committee. All participants were informed and gave their written consent.

The population included in the study were 19 patients older than 18 years, treated at the General Surgery and Digestive System Service of the OSI Bilbao-Basurto, who have undergone an ostomy (colostomy, ileostomy or urostomy) with more than six months after it had been performed. Subjects with intellectual disability or cognitive impairments were excluded. 19 of the patients met the inclusion and exclusion criteria. Table 1 summarizes baseline characteristics of these patients.

### 2.2. Hospital Visits and Scanning Protocol

**Figure 2:**
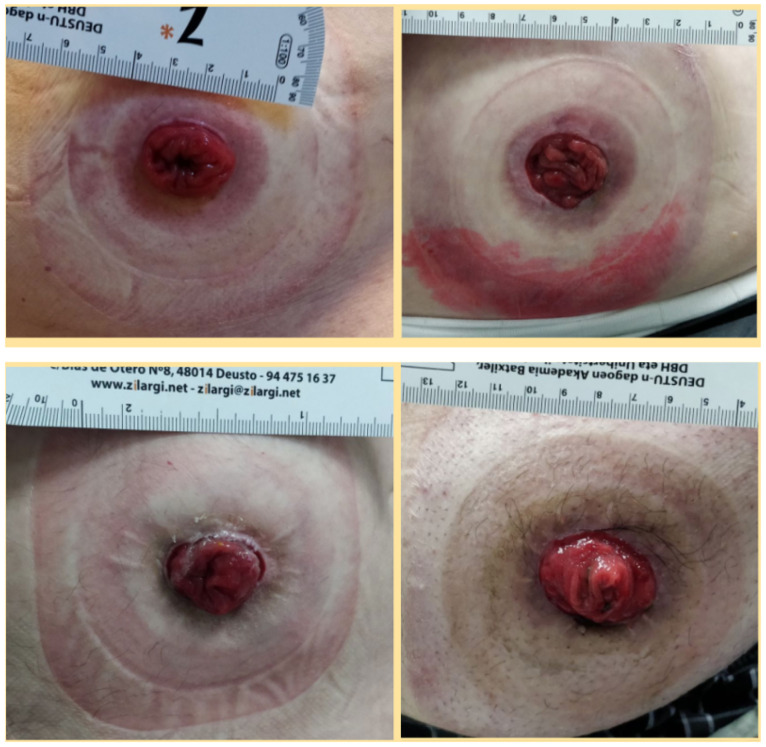
Collage of some photographs of the scanned stomas during the hospital study visits.

In order to acquire the 3D scans of the abdominal regions and assess the option of using 3D scanning systems which are considered a low-cost alternatives to conventional scanning techniques in systems health, 19 patients were selected and agreed to have their abdominal area around the stoma scanned. The patient was first assisted in order to remove the patch on the stoma, in order to prepare the bare skin for the scanning. Using both the Structure Sensor and the Go!Scan scanners, the patient was asked to remain standing with minimal displacement for a better scanning accuracy. The average time spent with a single patient was around 30 min, including the removal of the first stoma patch, and dressing the new one after the scanning was completed, as shown in Figure 2.

### 2.3. Data Acquisition

In our study, we used the 3D scanning devices Occipital Inc. Structure Sensor (ST01) [26], as well as the Creaform Inc. Go!Scan 20, in order to acquire the 3D meshes of the surfaces around the ostomies.

#### 2.3.1. Occipital Inc.: Structure Sensor

**Figure 3:**
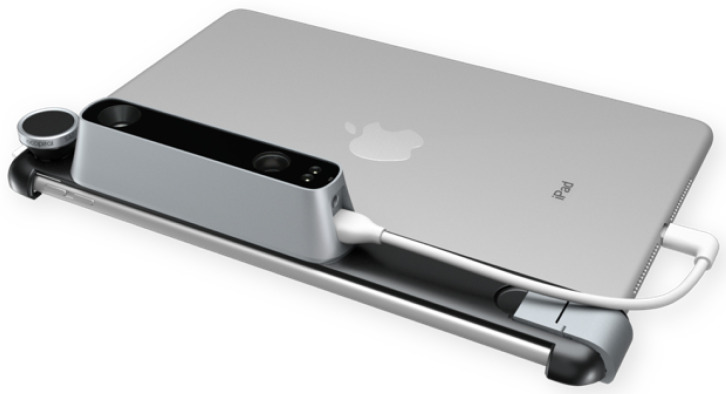
The Structure Sensor mounted on an iPad.

The Structure Sensor is the first 3D scanner used on mobile devices. This Structured Light System (SLS) contains a laser-emitting diode, an infrared radiation range projector, and an infrared sensor. Then, using a safe infrared light, the sensor scans the objects and the iPad’s RGB camera sends data to a System On a Chip (SOC) for processing. The Structure Sensor is a software controlled scanner and can be mounted on an iPad with its customized bracket and works on a rechargeable battery, as shown in Figure 3.

The sensor alone delivers a point dataset, of a 640 × 480 pixels resolution, where each pixel contains the distance from the sensor to the target. The role of the infrared (IR) sensor is to record the reflectance intensity of the infrared light pattern projected by the IR projector onto the target, then the point cloud is triangulated on the PrimeSense SoC to form the 3D mesh [27].

**Figure 4:**
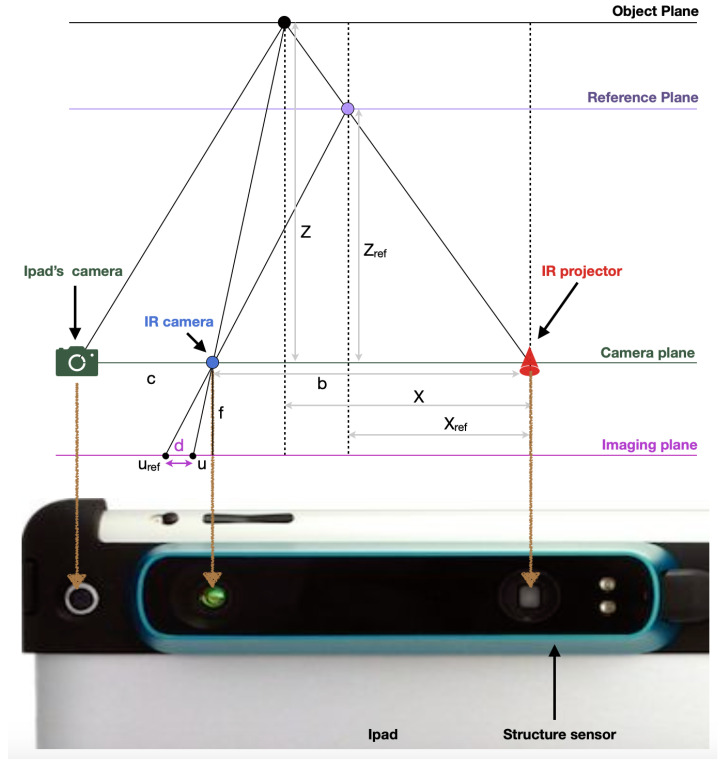
Explanation of the geometry of converting disparity to depth used by the Structure Sensor (adapted from the work in [28]).

During the acquisition of the 3D scene, the target point, shown as a black point in Figure 4, is projected at depth Z from the camera plane. The IR camera is situated at a distance b = 65 mm from the IR projector, whereas the iPad’s RGB camera is situated at a distance c = 6.5 mm from the IR camera. Depth images are constructed on the imaging plane through the perspective IR camera. Let us assume that an object on the reference plane is at a distance Zre f to the sensor. If the object is shifted closer or further from the sensor, a displacement in pixels between the two patterns is created on the imaging plane, called the disparity (Equation (1)):

(1) $$d=u-u_{ref}$$

Using the trigonometry relations using the triangles in Figure 4, and by determining the constant parameters $Z_{ref}$ and the focal length *f*, using calibration, the depth *Z* can be obtained using the following equations (Equations (2)–(4)):

(2) $$\frac{X-X_{ref}}{b}=\frac{Z-Z_{ref}}{Z}$$

and

(3) $$\frac{d}{f}=\frac{X-X_{ref}}{Z_{ref}}$$

(4) $$Z=\frac{Z_{ref}}{1+\frac{Z_{ref}d}{fb}}$$

#### 2.3.2. Creaform Inc. (Academia): Go!Scan 20

**Figure 5:**
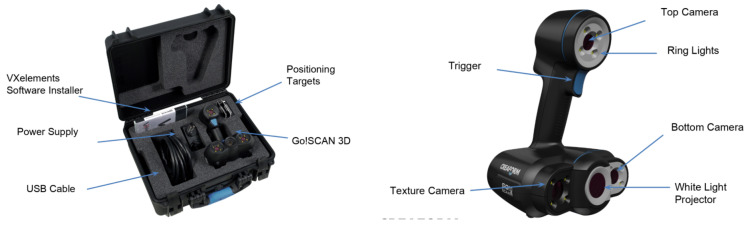
(**Left**): Go!scan 20 package, (**right**): Go!scan 20 3D components.

Go!SCAN 3D requires geometry in order to position itself. The white light pattern is projected by the LED onto the object. The pattern distortion on the object is recorded by the two digital cameras: one camera is placed on top of the scanner and the other one is placed on the bottom right-hand side when facing the scanner. The acquisition is made over the entire light pattern. The geometry information collected is used to build the surface in real-time positioning, as shown in Figure 5. The scanner also works with an intelligent hybrid positioning method which requires installing some positioning targets on the surface to be scanned. The scanner combines the positioning targets provided with the geometry information in order to provide more accurate results. Texture positioning could also be combined with the aforementioned methods in order to get optimal results. The scanner acquires and detects the object texture with its digital color camera.

**Figure 6:**
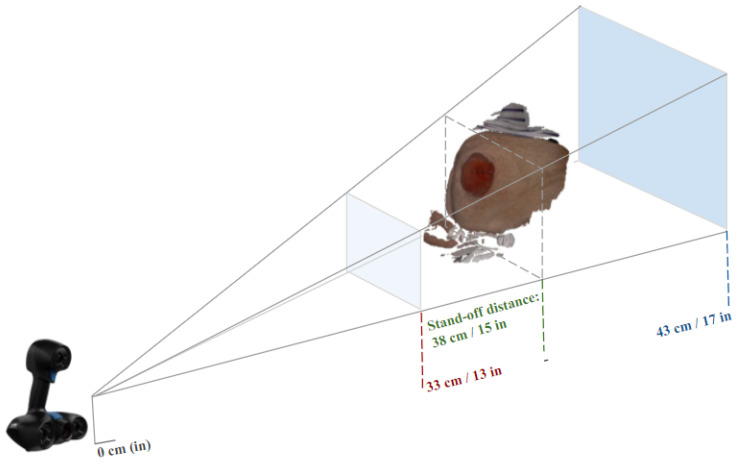
Illustration of the required distance to surface for the Go!scan 20.

When scanning from the proper stand-off distance, Go!SCAN 20 projects a smaller light pattern. This scanner provides better surface resolution and accuracy, resulting in a much more detailed capture. For optimal field of view, stand-off distance should be 380 mm. Working distance should be between 330 mm and 430 mm, as shown in Figure 6.

#### 2.3.3. Technical Comparison between the Structure Sensor and Go!Scan 20

The characteristics of the handheld 3D scanners used in this study are presented in the following Table 2.

#### 2.3.4. CT Scans

**Figure 7:**
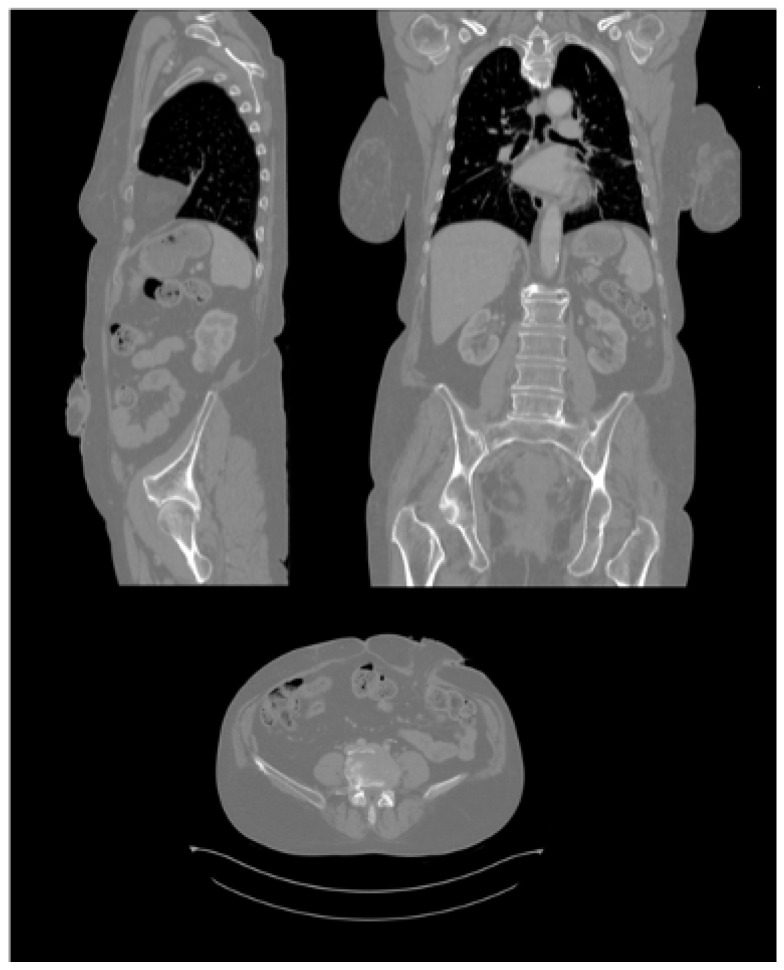
Cross sectional images (sagittal, coronal and axial) of an abdominal CT scan [31].

To create the three-dimensional model of the abdominal geometry of the patients, which would allow the design and printing of the molds of the personalized ostomy meshes, medical images retrospectively obtained by computed axial tomography “CT scan” were obtained from the 19 participating patients. The Statistical Information Unit of the U. Basurto Hospital provided the CT scan images files encoded and in “DICOM” format. CT scan is a classical medical image acquisition method. This imaging technique is based on computed processing of multiple X-ray measurements. This allows to have a fast 3D representation of the body of the patient in a minimally invasive manner. The technique consists of a narrow beam of X-rays is aimed at a patient and quickly rotated around the body, producing signals that are processed by the machine’s computer to generate cross-sectional images—or “slices”—of the body [30]. This technique is performed by means of a CT scanner, a high cost equipment which requires expertise for adequate usage. Each complete human body acquisition takes around 20 min, and the patient must stand still in order to minimize detection errors (noise) during the image sampling. Figure 7 shows the three cross sectional images of an abdominal CT image.

#### 2.3.5. Processing of CT Scans and Design of the Ostomy Patch

For the image processing step, a software compatible with DICOM type files is required. For this study, the chosen software was 3D Slicer [32], a free software for multimodal medical image processing compatible with CT, MRI (Magnetic Resonance Imaging) or US (Ultrasound) scans.

**Figure 8:**
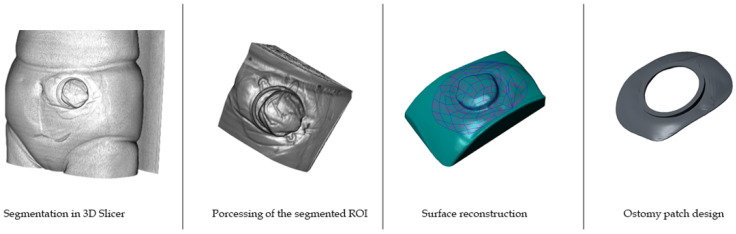
Main steps for the design of the personalized ostomy patch via CT scans.

CT scans containing a full 3D representation of the thoracic part of the patient were received from the medical personnel. Steps to be followed for the design of the ostomy patch are summarized in Figure 8.

**Figure 9:**
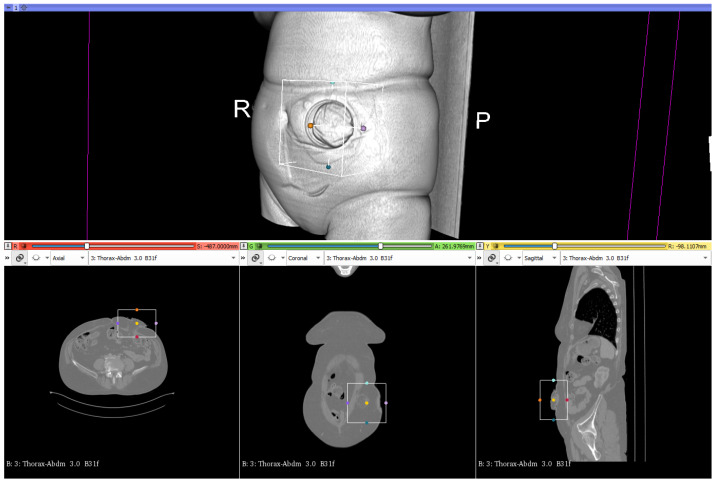
Selection of the stoma region in 3D Slicer.

First, the images need to be segmented and processed to select and crop the region of interest (ROI), the abdominal region surrounding the stoma. After file importation to the software, the ROI is displayed, selected and segmented with an easy and precise method using the 2D and 3D visualization methods, as shown in Figure 9.

**Figure 10:**
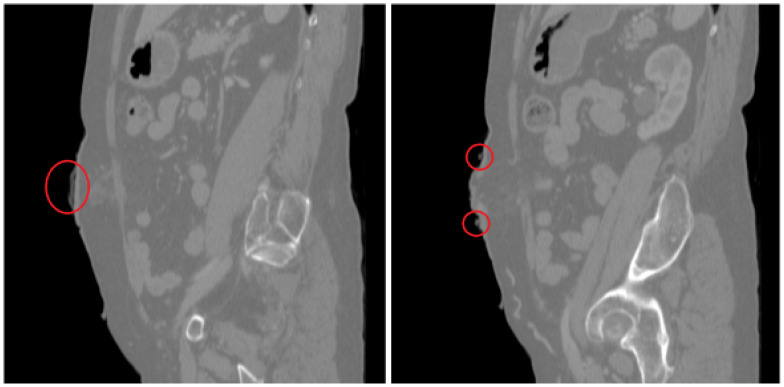
Profile of the segmented ROI (red circle points the patch connector).

Regarding CT scanning of ostomy patients, they are usually asked to wear the ostomy patch and connector during the scan due to hygienic reasons and the time needed by the patient to take it off. Therefore, the scanned ostomy patch connector must be erased from the ROI in order to obtain a clean abdominal surface geometry, as shown in Figure 10 (profile view of the stoma connector).

**Figure 11:**
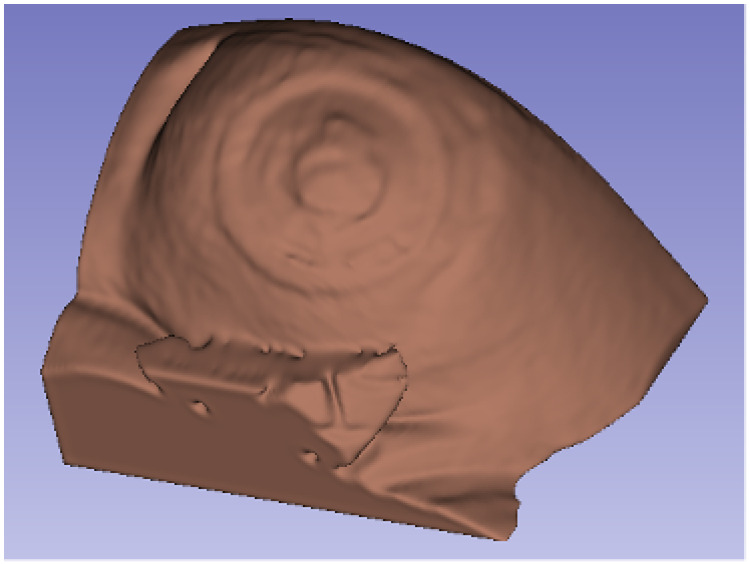
Final 3D volume of the ROI obtained by 3D Slicer.

The Threshold command allows to select a specific range of grayscale intensities from the 3D image. This way, different tissues can be filtered, selected, and erased. In this case, the threshold value which corresponds with the abdominal skin was applied. Manual corrections are usually required after this step to completely erase the connector. Finally, the 3D volume of the abdominal skin is obtained, which contains the information of the ROI (i.e., the region of the stoma and its surroundings), as shown in Figure 11.

From this point on, the design steps to be performed in Creo Parametric are the same for the CT scans as for those obtained by the Structure Sensor or Go!Scan 20 scanners.

In a second stage, the obtained surface, regardless the acquisition equipment, is imported to a CAD software. For this study Creo Parametric [33] from PTC Inc. was used to design the personalized ostomy patch and the mould to be 3D printed for medical grade silicone. The patch must perfectly fit with the specific geometry of the abdominal surface of the patient to achieve the best possible adhesion and comfort.

**Figure 12:**
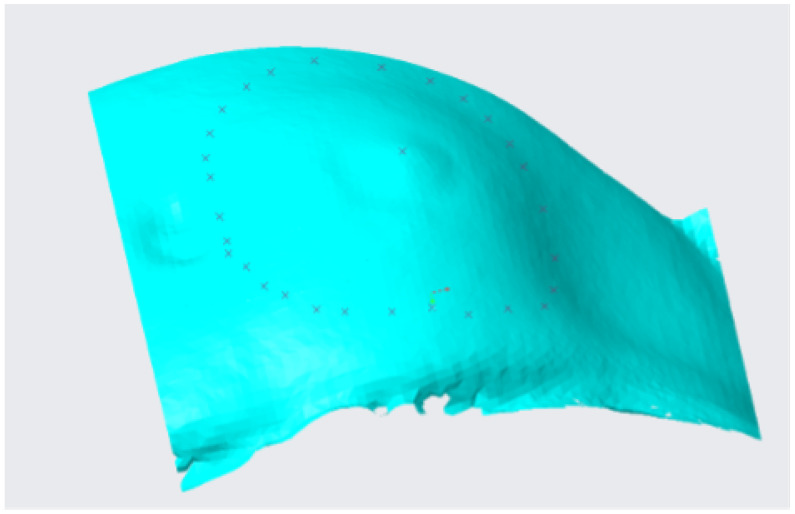
Points marking on the STL file the perimeter of the patch and stoma location.

Creo Parametric is a robust modeling tool which gives an instant feedback for design changes. It provides surface capabilities to build or organically shape complex surfaces, which is needed for the process of generating the personalized ostomy patch. Stereolithographic (STL) format files are usually used to be exported from 3D Slicer into Creo Parametric, which allows full compatibility between the two softwares. However, direct edition of this type of surface file is not possible. Thus, the first step of the design process consists of generating an editable surface from the imported geometry. This is performed by surface reconstruction operations. The quality and resolution of the surface reconstruction depends mostly on the geometry and resolution of the imported STL file. This type of files is based on a mesh composed by adjacent triangles. In the reconstruction step, the software needs to be capable to join all the triangles surfaces to generate a unique quilt, which will constitute the skin/contact surface for the personalized ostomy patch design. Taking the stoma as a reference point, the perimeter of the patch is defined, as shown in Figure 12.

**Figure 13:**
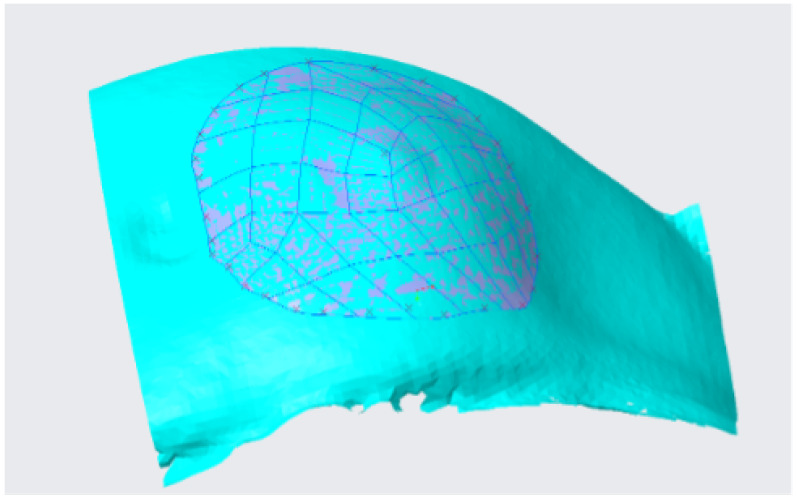
Quilt generated after the reconstruction process.

A quilt is generated in the reconstruction step, which can be discretized with surfaces of different geometries, as triangles, rectangles or pentagons. Figure 13 shows a representative quilt generated by rectangular patches. Usually, the generated quilt needs to be analysed to verify that all the rectangular patches form a continuous surface, as this is necessary for a correct reconstruction.

**Figure 14:**
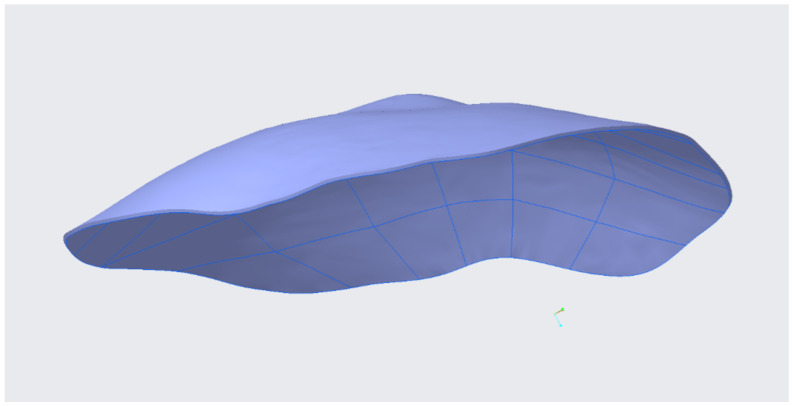
Design of the personalized ostomy patch.

Finally, an offset operation was applied to the quilt to give it the desired thickness. In this study, a constant thickness of around 1 mm was applied, which is close to the thickness of the commercial flat patches. This way, the design of the ostomy patch which contains the specific geometry of abdominal surface of each patient is obtained, as shown in Figure 14.

### 2.4. Data Processing

**Figure 15:**
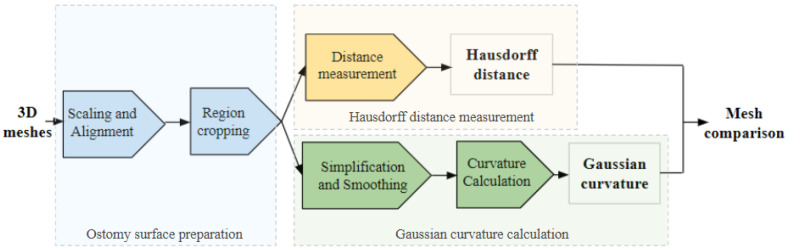
Diagram of mesh processing steps.

The meshes acquired from both sensors were in .obj format. The software which was used to process the meshes was Meshlab, which is an open source system for processing and editing 3D triangular meshes. This software provides several tools for editing, cleaning, inspecting and rendering meshes, and features for processing raw data in order to prepare them for 3D printing. The processing framework which has been developed in order to compare the meshes is composed of different steps, as shown in Figure 14. First of all, the meshes were scaled to the same millimeter scale, and aligned using Iterative Closest Point (ICP) algorithm [34]. Then, the meshes were cropped in such a way that solely the area of interest around the ostomy is selected. The first comparison method chosen was Hausdorff distance, in order to compute the distance between each point from a reference mesh to the closest point from the other mesh, as shown in Figure 15.

#### 2.4.1. Ostomy Surface Preparation

Once the meshes were imported in the software, the first step was to scale both meshes in the same millimeter scale in order to have the same real world measurement. Then, the alignment was firstly handled by gluing one mesh and selecting the point based gluing. In this step, both meshes are shown and at least 4 corresponding points in each mesh are selected manually. This step will help the automatic alignment later on. After applying the point based gluing, the Iterative Closest Point (ICP) algorithm was applied in order to affine the alignment.

The ICP algorithm is a method used to align two points set with each other, by finding the correspondences between the points. Whether it is to solve a rigid transformation, scale or affine, the ICP algorithm finds the transformation between the two sets using an iterative search method. Let us assume that the points of the model X are $X_{i}|X_{i}\in R^{3},i=1,2,\ldots ,N_{X}$ and the point of the target model Y are $Y_{i}|Y_{i}\in R^{3},i=1,2,\ldots ,N_{Y}$. The aim is to find the rotation and translation matrices R and t by minimizing the following cost function: $E\left(R,t\right)=\sum_{k=1}^{N_{Y}}{∥X_{k}-RY_{k}-t∥}^{2}$. The steps of the ICP algorithm are the following:

- Finding the nearest point for each point pair on the reference model.
- Estimating the rotation and translation matrices R and t such that $\sum_{k=1}^{N_{Y}}{∥X_{k}-RY_{k}-t∥}^{2}=min$.
- Iterating through the previous steps until the error is less than a given threshold value or the maximum number of iterations is reached [35].

**Figure 16:**
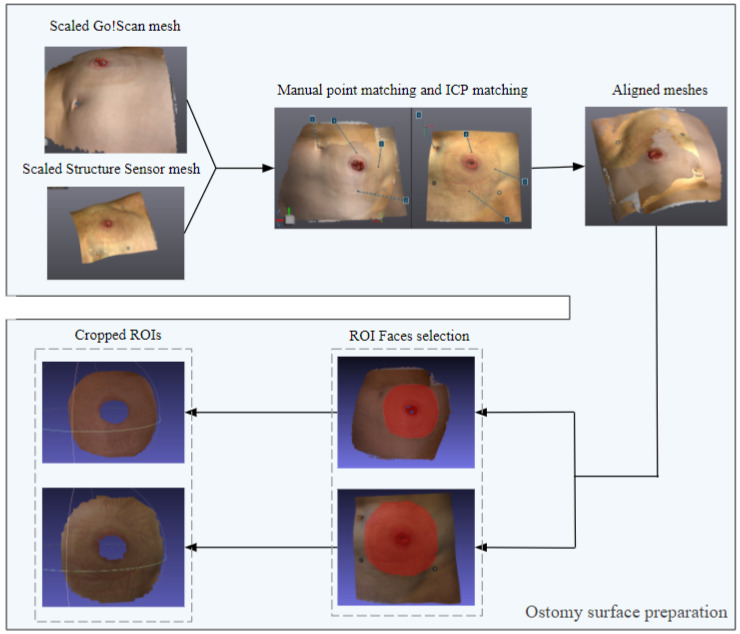
Stoma surface preparation illustration.

Because the ICP algorithm is sensitive to local minimas, a preliminary manual matching was made in order to bring the meshes to a closer distance from each other. Once the meshes were aligned, the surface around the stoma was manually cropped in both meshes, using the face selection and deletion function, as shown in Figure 16.

#### 2.4.2. Hausdorff Distance Measurement

Hausdorff distance is the maximum distance of a set to the nearest point in the other set. More formally, Hausdorff distance from set A to set B is a minimum function, defined as: h(A,B)=maxmind(a,b) where a and b are points of sets A and B, respectively, and d(a,b) is any metric between these points [36]. For simplicity, we will take d(a,b) as the Euclidean distance between a and b. This means that in order to compute the Hausdorff distance, first the nearest point in B for every point in A is found, and then the largest of these values are taken as the distance, which represents the most mismatched point of A. Using this metric, we can estimate how close the meshes are after having them aligned.

#### 2.4.3. Gaussian Curvature Calculation

In order to calculate the curvature of the meshes, we applied firstly a simplification of the mesh from the Go!scan in order to reduce the number of faces to the same number as the mesh from the Structure Sensor. For this simplification, we used the Quadric Edge Collapse Decimation method [37], available in Meshlab. Then both meshes were slightly smoothed using Taubin smoothing function [38]. Curvature values can be used to shade the surface of a mesh to gain insight as to where the surface is curvier. One definition for curvature is the magnitude of a change in angle with respect to a change in arc length. When there is a change in the angle at some point, the arc is deemed to be curvy at that point [39].

## 3. Results and Discussion

The fundamental contribution of this study is that it has shown that it is possible to use low-cost scanners to obtain images of the abdominal surface without irradiating the patient, reducing the risks of new cancers due to radiation emitted by conventional methods. In addition, it would reduce the discomfort of patients with surgical procedures or treated for colorectal cancer who undergo numerous scanner evaluations throughout their follow-up, due to the recurrences of their original tumors.

**Figure 17:**
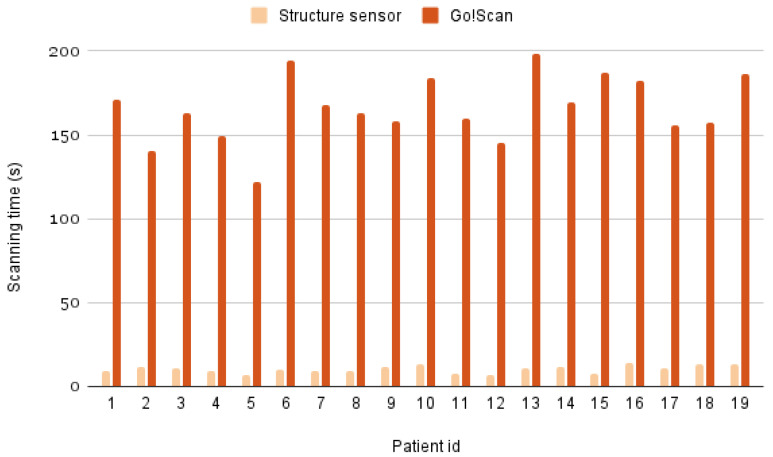
Scanning time taken using the Structure Sensor and the Go!Scan for each patient.

As for the comparison between the meshes acquired using the Structure Sensor and the Go!Scan 20, the following differences were found:

In terms of mobility and time efficiency, the Structure Sensor acquisition was almost 20 times faster than with the Go!Scan, with an easier mobility and handling. The Go!Scan needs to be connected to the laptop during the scanning in order to be able to follow the scanning and adjust it when necessary. Figure 17 shows the scanning time taken by each scanner for all the patients.

**Figure 18:**
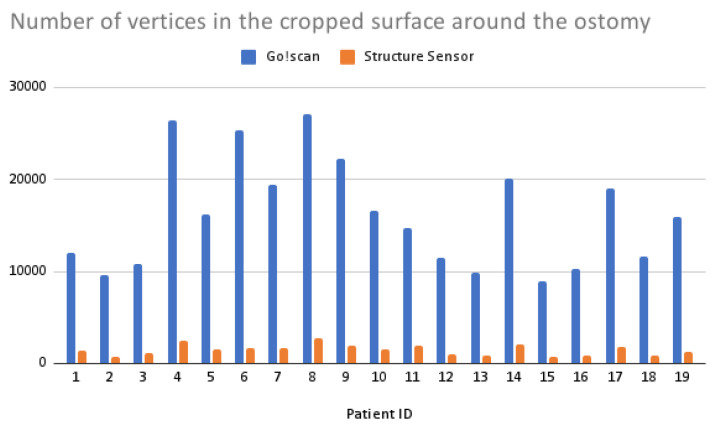
Bar plot of the number of vertices in the cropped surfaces acquired using the two scanners.

**Figure 19:**
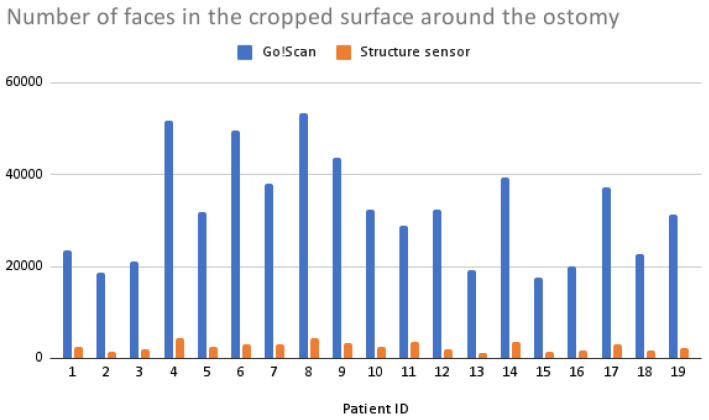
Bar plot of the number of faces in the cropped surfaces acquired using the two scanners.

In terms of 3D reconstruction reliability, the Structure Sensor gives a complete closed mesh in a matter of seconds, while the color definition and vertex number is lower compared to the Go!Scan’s, as shown in Figure 18 and Figure 19, whereas the Go!Scan gives a very defined mesh and color information, especially when the target stickers are placed on the skin surface. However, it needs a trained user to maintain the correct distance and angles, otherwise the scanning position could be lost, and very often the scanning is to be repeated.

**Figure 20:**
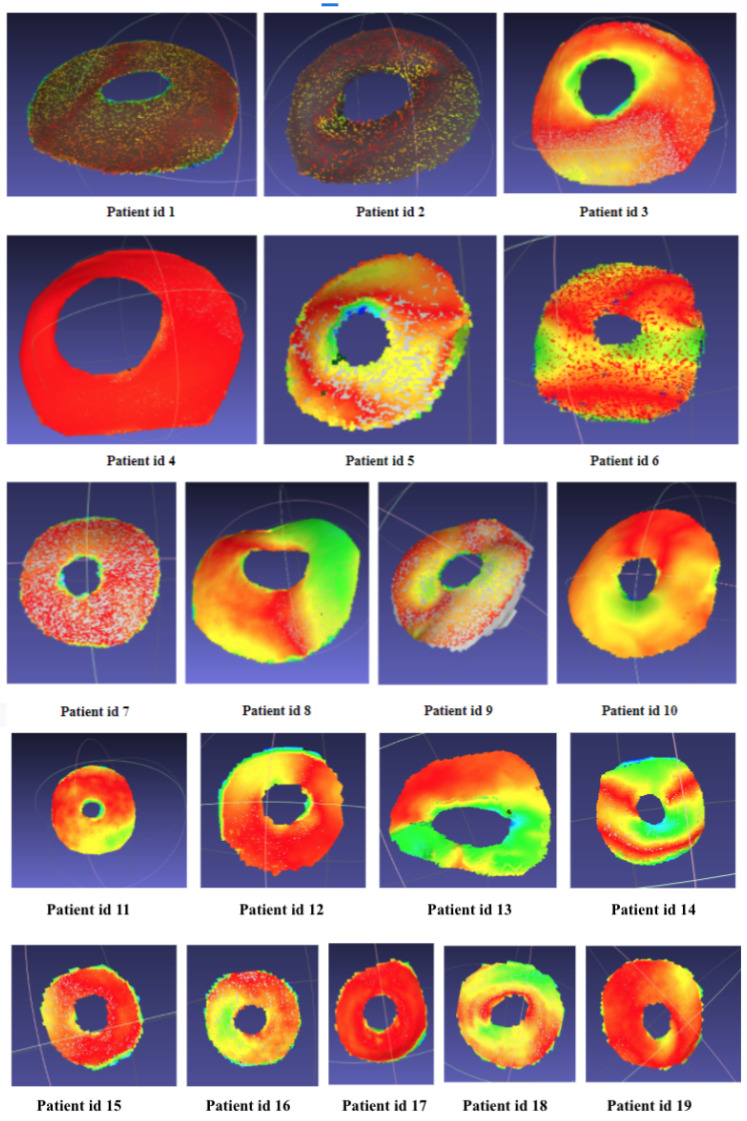
Collage of the Hausdorff distances of the 19 pairs of meshes.

**Figure 21:**
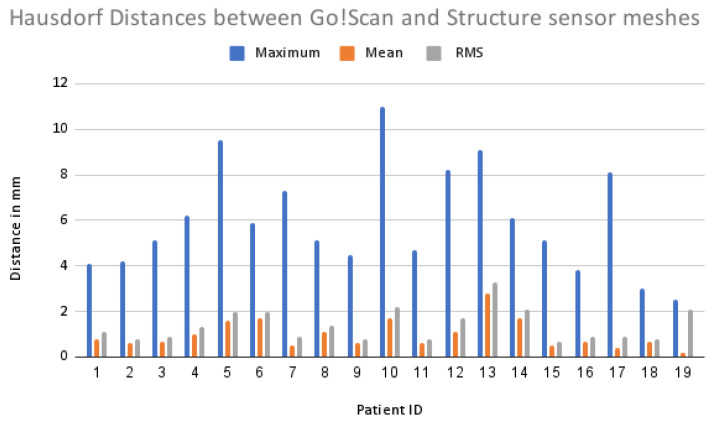
Bar chart of the mean, maximum and RMS error using the Hausdorff distances.

From the results obtained using the calculation of the Hausdorff distance, we can notice that even though the precision of the Structure Sensor is lower than the Go!Scan’s, the surfaces are very close, as shown in Figure 20, where the mean value of the distances between the meshes in all the patients is around 1.03 mm and the average Root Mean Square error is 1.34 mm, as shown in Figure 21.

**Figure 22:**
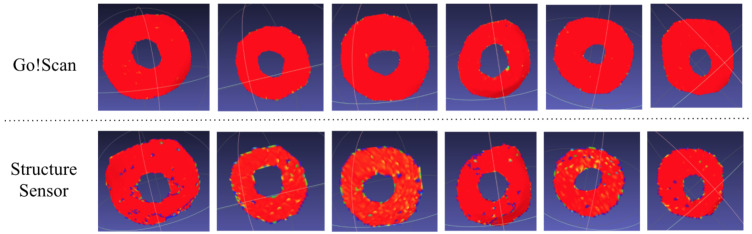
Examples of gaussian curvature calculation for surfaces acquired using the Go!Scan 20 and Structure Sensor.

In addition to this, the Gaussian curvature confirmed the obtained results, as the surfaces from the Go!Scan were very smooth, and those with the Structure Sensor had very slight curvatures, which did not have any impact on the printed patches, as shown in Figure 22.

A clinical study was designed to assess the safety and functionality of the ostomy devices designed and prototyped for the project, which included a total of 19 patients of the General Surgery and Digestive System and Urology Services from the Basurto University Hospital (Basque Health System, Osakidetza). Among other assessed items concerning the device, the customised ostomy patches were tested in nine of the 19 patients in the study, with a total of 22 patches being delivered and tested.

All observations made by the patients and incidents related to the patches were recorded. The assessed variables included (a) date on which the customized patch was fitted to the abdominal surface; (b) date of disc detachment; (c) type of scanner with which the surface was scanned for each patch; (d) reason for the detachment of the patch (lack of adhesion, itching/burning, peristomal dermatitis or other); and (e) feedback related to patch orientation, abdominal fit, adherence to skin, perceived comfort compared to commercial patches.

Problems reported by the patients were mostly related to a poor adhesion of the disc to the skin. Patch rupture was reported in just one case, and skin irritation in another case. There were also complaints about the thickness of the patch when compared to the commercial one. Most of the patches allowed proper use for 3 to 12 h, and only one patient reported that the patch remained in place for more than 3 days. The feedback received from the patients during the clinical tests showed no difference between the patches designed from each scanner. In terms of reliability of the resulting mesh, the little differences on resolution of each equipment are not noticeable in the final patch. This is mainly due to two reasons: on the one hand, the 3D printing technology used for the manufacture of the molds (FDM) causes a slight surface roughness, which homogenizes the resulting qualities. The stoma patch is impregned with a skin-contact medical-grade adhesive silicon (for the adhesion onto the skin), which again evens out any small differences in the inner surface of the patch.

In terms of scanning methodology, the Structure Sensor requires solely the definition of the volume containing the surface to be scanned, which can be easily done by zooming in or zooming out the cube on the screen using the fingers. Then, the scanning is quickly made by moving around the surface. As for the Go!Scan, the scanner comes with a software called VXmodel, which is needed for the scanning. This software needs previous learning to understand the acquisition methodology. Several parameters can be changed, which affects also the definition of the resulting mesh. The position of the scanner from the surface is very important (distance and angles), otherwise the scanner loses its position on the mesh, and finding it again is often delicate. Therefore, the Structure Sensor proposes an easier and effortless scanning methodology compared to the Go!Scan 20.

**Figure 23:**
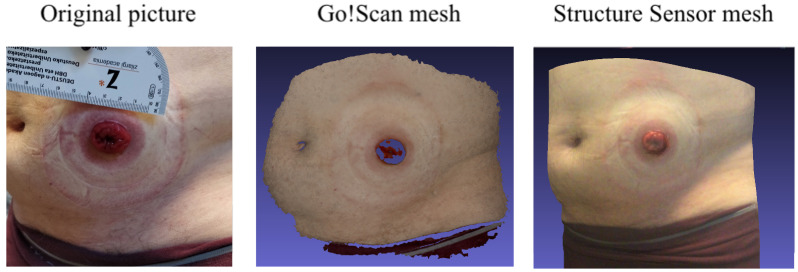
(**Left**): Original picture of the ostomy. (**Middle**): The mesh acquired using the Go!Scan. (**Right**): The mesh acquired using the Structure Sensor.

In terms of influence of lighting conditions and object materials, the Structure Sensor here again offers a wider ranger of materials and colors to be scanned. In fact, the Go!Scan cannot scan materials with low reflectance and gloss specularity. For instance, both scanners were used to attempt to scan a person’s black hair, and only the Structure Sensor was able to deliver the mesh. As for the stomas, when the surfaces were covered with a liquid, the Go!scan could not detect them, as shown in Figure 23.

As for the comparison with the CT scan, The process of designing a personalized ostomy patch by means of a manual scanner such as Structure Sensor or Go!Scan 20 presents several advantages compared to the CT scan. Regarding the patient, the handheld 3D scanning method opens the possibility to acquire the abdominal surface data anywhere, hence avoiding the need of an appointment with the Hospital. This leads to reducing expenses and saving time due to the use of less expensive equipment and a reduced workload for healthcare personnel. Furthermore, from an engineering point of view, working with the files obtained from the manual scanners has been shown to be faster (less complex) than with TC DICOM files. The process of erasing the stoma connector was avoided when using the manual scanners, as they allow to acquire bare abdominal surfaces. This also helps to achieve better results as the process of eliminating the connector is somehow subjective and causes little errors on the abdominal surface to be used to design the patch. In addition, handheld 3D scanner archives are lighter than CT scans data, what gives the possibility to work faster in the design software.

**Figure 24:**
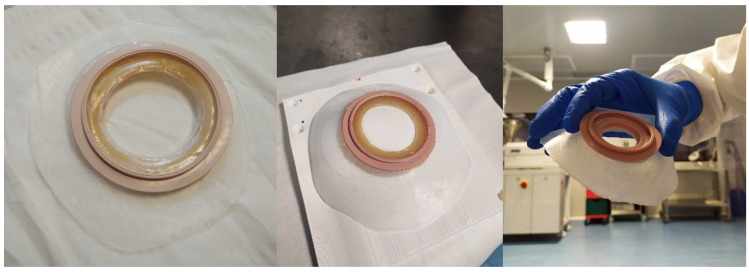
The three stoma patches produced for a patient of the clinical trial. From left to right, using CT scan, Structure Sensor and Go!Scan 20.

As a final result, comparing the final ostomy patches that have been produced starting from the 3 different types of images (CT scans, Structure Sensor and Go!Scan 20), there is not any difference at either big or small scale. The roughness of each patch remains the same, as mentioned previously, because it is determined by the process of 3D printing. In Figure 24 are shown the three final stoma patches that were produced for the same patient, using the three types of medical images.

## 4. Conclusions

In this paper, we presented a comparative study of 3D scanners for a personnalized 3D printing of stoma barrier rings. Two different scanning modalities have been used: the structured light scanning and CT scans. In the structured light scanning, two devices have been compared: the Structure Sensor (ST01) from Occipital Inc., and the Go!Scan 20 from Creaform Inc. Comparing both sensors has led to the conclusion of the adaptability of the Structure Sensor for the scanning of the stomas as it outperforms the Go!Scan in several points, such as mobility and time efficiency, size obstruction, simplicity of scanning, range of scanned materials and reduced price. As for the CT scans methodology, it has proven to be not only more complex in the scanning protocol but also in the DICOM files processing. Hence, the handheld 3D scanners present more advantages compared to the CT scans in such medical applications.

## Figures and Tables

**Table 1:** Characteristics of the population.

Characteristics	N	Parameter
**Weight (kg)**	19	-
Median (P25–P75)		77 (69–84)
**Age (years)**	19	-
<45	2	-
45–54	3	-
55–64	7	-
65–74	7	-
Median (P25–P75)		61 (53–67)
**Gender (Male)**	13	68.40%
**Height (cm)**	18	-
Median (P25–P75)		168.5 (163.7–174.0)
**Pathology of origin**	19	-
Rectal neoplasm	6	31.60%
Colon cancer	1	5.30%
Radical cystectomy for bladder tumor	2	10.50%
Recurrent interstitial cystitis	1	5.30%
Ulcerative colitis	2	10.50%
Crohn’s disease	4	21.10%
Anal malformation and Crohn’s disease	1	5.30%
Bladder neoplasm	2	10.50%

**Table 2:** Technical comparison between the Structure Sensor [26] and the Go!Scan 20 [29].

	Structure Sensor	Go!Scan 20
**Operating range (recommended)**	0.4–3.5 m	0.05–0.5 m
**Accuracy**	0.5 mm at 40 cm 30 mm at 3 m	Up to 0.1 mm
**Mesh resolution**	1 mm	0.100 mm
**Weight**	95 g	930 g
**Dimensions (L × W × H)**	29 × 28 × 119.2 mm	154 × 178 × 235 mm
**Light source**	Infrared LEDs	White LED
**Output Formats**	.obj	.dae, .fbx, .ma, .obj, .ply, .stl, .txt, .wrl, .x3d, .x3dz, .zpr
**Price**	$379 without mounting device	Around $10,000 with the scanning software only (VXmodel)

## Abbreviations

The following abbreviations are used in this manuscript:

SLS	Structured Light System
SOC	System On a Chip
IR	Infrared
CT	Computed Tomography
